# The Family Regulation System and Medical-Legal Partnerships

**DOI:** 10.1017/jme.2024.2

**Published:** 2023

**Authors:** Kara R. Finck, Susanna Greenberg

**Affiliations:** 1: UNIVERSITY OF PENNSYLVANIA CAREY LAW SCHOOL, Philadelphia, PA, USA; 2: HELP: MLP, PHILADELPHIA, PA, USA

**Keywords:** Family Regulation System, Medical-Legal Partnerships, Child Welfare System, Child Advocacy, Interdisciplinary Practice, And Nurse Partnerships

## Abstract

This article confronts the challenges and opportunities presented by medical-legal partnerships (MLPs) representing families impacted by the family regulation system. Based on the authors’ experience developing a collaboration between a medical-legal partnership, interdisciplinary law school clinic and nurse home visiting program focused on clients impacted by the family regulation system, the article challenges traditional conceptions of the MLP model and proposes an expanded vision for MLPs to address systemic injustice and improve outcomes for families.

Through years of working to address the health and legal needs of pregnant and parenting women as a medical-legal collaborative of nurse home-visitors, lawyers, and social workers, we have witnessed firsthand the impact of the child welfare system on our clients who are pregnant or parenting their first child. Clients alternate between hope and anxiety, filled with love for their children but worried about how they will prevent their infants from entering an unjust system. That concern is well founded since threats to their family autonomy flow freely from routine interactions with daycare staff, benefits caseworkers, landlords, and medical personnel, who often threaten reporting a mother to child welfare officials to compel her compliance.

Examples include a shelter caseworker calling in a report of suspected maltreatment against a young mother in foster care because she lacked formula for her infant daughter, disregarding the fact that there was a national shortage of infant formula. Another client requested support in scheduling a C-section or finding alternate care for her first child, only to be informed by her doctor that a report would be called in if she could not provide a definitive plan of childcare despite the inherent unpredictability of labor and delivery. A nurse home visitor appeared in family court to attest to her client’s caregiving, which she personally witnessed every time she visited the young family in their home, only to be swiftly informed that she was not a party to the proceeding and had to remain outside of the courtroom, instead of beside her client. Time and time again, even when a client is enrolled in our partner nurse home visitor programs, with trained medical professionals experienced in infant and child development, clients lack any shields to protect them from institutional scrutiny of their parenting and corresponding threat of separation from their child.

In this paper, we propose that the medical and legal professions, specifically through the medical-legal partnership (MLP) model, can and should play a critical role in dismantling the child welfare system and its legacy of inflicting trauma on families of color by providing children and families with supports that protect child safety and promote family well-being.

Medical-legal partnerships, which seek to empower families, address structural racism, and promote child well-being, must engage substantively with the child welfare system and the movement to transform it from a system that regulates families, particularly families of color. Medical and legal professionals played critical roles in the development of the current child welfare system by focusing on expansive definitions of neglect under the law, mandated reporting, and a historical preference for child separation at the expense of family preservation.^1^ In this paper, we propose that the medical and legal professions, specifically through the medical-legal partnership (MLP) model, can and should play a critical role in dismantling the child welfare system and its legacy of inflicting trauma on families of color by providing children and families with supports that protect child safety and promote family well-being.

Our collaboration between a nursing-legal partnership and an interdisciplinary child advocacy law school clinic, demonstrates the promise for how MLPs can address the trauma and racial disproportionality of the child welfare system, now more commonly referred to as the family regulation system.^2^ Dr. Barry Zuckerman, the founder of the MLP movement, underscored the importance of this shift in focus when he advocated for a two-generation approach to pediatric care, noting that “the best way to help children is to help their parents, and the best way to reach parents is through their children.”^3^ MLPs provide a transformational opportunity to reframe how children and families receive services, address social determinants of health, and create anti-racist systems that support family autonomy and engagement. The MLP movement can expand its mission to address the social and structural determinants of health by learning from the growing body of research on the impact of the family regulation system on child and family well-being and actively engaging with clients involved in the family regulation system.

Over the past three decades, medical-legal partnerships transformed the delivery of legal services, enhanced interdisciplinary practice by incorporating a variety of medical professionals into the legal team, and reframed the identification of legal issues by focusing on screenings for the social determinants of health.^4^ Nationally, MLPs address a wide range of legal issues — including traditional civil legal services such as housing, benefits, and utilities — while also expanding the model to address the needs of specific client populations, such as patients with chronic illnesses, veterans, and seniors.^5^ MLPs in pediatric care settings have not grown as quickly or expansively as other partnerships: there are only 37 in children’s hospitals.^6^ And by design, pediatric MLPs often do not include families impacted by the family regulation system, due to concerns about mandated reporting, representation in dependency proceedings, and questions around whether the MLP’s legal representation attaches to the pediatric patient (child) or their parent(s). In some MLPs, children and parents involved in the family regulation system are explicitly excluded from receiving legal services in the MLPs memorandum of understanding between the healthcare provider and legal services partners based on an implicit belief that including such cases would raise conflicts with the healthcare provider.^7^

In order to maximize the potential of MLPs to propel reform, we argue that individual MLPs and the larger health and legal systems must not revert to traditionally siloed practices focused on the protectionist mechanisms of the family regulation system (reporting, supervision, and removal) while simultaneously precluding parents from accessing the legal, medical, and social services and advocacy that would strengthen their families and are the hallmarks of MLP practice. The tendency of MLPs to exclude families impacted by the family regulation system limits the model’s ability to serve all families regardless of race and to promote anti-racist systems given the documented racial disproportionality of families involved in the family regulation system. Given the overlap between the social determinants of health and neglect as currently defined by the family regulation system, the role of MLPs that focus on children and families with actual or potential involvement in the family regulation system is critical. In Part I, we describe this overlap between the social determinants of health and neglect and the role of preventive and interdisciplinary representation in improving outcomes for families, highlighting our collaboration as a model for MLPs targeting these issues. In Part II we discuss the concerns that have been raised about MLPs in this context and a model for addressing those issues. Finally, in Part III, we advocate for the MLP movement to disrupt traditional legal and medical practice by embracing representation in these cases and increasing advocacy for preventive legal representation focused on partnering with and empowering families.

## I. Overlaps in Issues and Practice

The family regulation system refers to the laws, policies, government institutions, and private social service providers authorized to intervene in the custodial rights of parents and caregivers through a variety of means including mandated reporting, child protection investigation, removal of children from their parents’ care and termination of parental rights. Nationally, “[i]n 2020, three of every four children that the child protection system deemed victims of child maltreatment fell into the category of neglect, not abuse.”^8^ Neglect involves a range of issues including inadequate housing, lack of access to childcare, food instability, educational neglect, substance abuse, and behavioral health. These issues are closely linked with poverty and the social determinants of health, which can be ameliorated through civil legal services. For example, a single mother would be at risk of a report for lack of supervision if her state subsidized childcare benefits were improperly denied, and she was faced with the choice of leaving her child unsupervised or losing her employment. A family whose landlord refuses to make repairs could face a report called in by the landlord if they withhold rent or a finding of inadequate and unsafe housing if a mandated reporter visits their home. Lack of a consistent treatment for mental health for either the parent or the child can lead to a report, even if the inconsistency is due to a lack of transportation, childcare or accessibility of appropriate mental health care.^9^ The family regulation system identifies these issues as neglect by the parent, triggering a report and range of interventions which can include the child’s removal from their parent(s). MLPs identify these issues as barriers to stability, health, and child well-being which have legal and non-legal remedies.

Pediatric MLPs generally focus on the traditional range of legal issues associated with the social determinants of health including eviction, special education services, and disability benefits.^10^ However, the work of two specific medical-legal partnerships with a focus on advocacy for mothers whose infants are born exposed to drugs offers a framework for assessing the potential impact of MLPs in this area. First, based in Washington State, the F.I.R.S.T. clinic offers legal representation prior to the filing of an abuse or neglect petition.^11^ The program succeeded in preventing a filing of a dependency case or removal of an infant in most cases by focusing precisely on the social determinants of health identified by traditional MLP screening efforts. Second, the University of New Mexico Medical-Legal Alliance FOCUS program serves pregnant and parenting mothers who used drugs during their pregnancy. If a mother or newborn tests positive for drugs, a referral is made to the program to provide legal representation and additional screening for health-harming legal needs and trauma.^12^

These models and their initial outcomes mirror research in traditional family defense representation that shows that interdisciplinary strategies are critical in addressing the unmet legal needs that lead to involvement in the family regulation system yet are not widely or readily available to families. The transformative role of legal representation during a child welfare investigation has been highlighted in previous literature^13^ and the significance of quality representation for parents in dependency court cases has been demonstrated unequivocally.^14^ The critical components of preventive lawyering include a multi-disciplinary partnership, community-based services, and legal representation, similar to the MLP model.^15^

Although most cases involve neglect resulting from underlying civil legal needs, parents’ access to quality legal counsel is severely limited and often entirely unavailable during the initial stages of a child welfare investigation. Legal services provided by MLPs can play a critical role in helping stabilize the family unit before, during, and after a period of removal, as parents continue to need support and counsel to address the legal needs that are construed by the family regulation system as neglect. The collaboration between Philadelphia Nurse-Family Partnership (NFP) and its sister program,^16^ HELP: MLP,^17^ and Penn Carey Law’s Interdisciplinary Child Advocacy Clinic (ICAC), grew out of this recognition of the potential of disrupting traditional pathways into the family regulation system and providing access to legal services at critical moments including child maltreatment investigations and dependency proceedings.

Our work focuses on addressing the health-harming legal needs of pregnant and parenting clients with current or former involvement with the family regulation system. At the start of the collaboration, NFP nurses implemented universal screenings for clients at regular intervals during their enrollment to identify legal issues and make referrals to their embedded civil legal aid partner, HELP: MLP. HELP: MLP attorneys assessed the legal issues identified and provided holistic representations for as many clients as possible, while referring more specialized issues to trusted partners. ICAC was the key partner to whom clients with family regulation system issues were referred for consultation and potentially representation.

It was critical to define the referrals to ICAC as broadly as possible, including pregnant clients wishing to re-enter foster care, parenting clients with a new or ongoing child welfare investigation, and clients who were discharged from foster care without critical support such as vital documents or housing. Additionally, cross-training focused on expanding the legal team’s knowledge of NFP’s core engagement strategies and teaching nurse home-visitors about the laws and processes of foster care and family court. As an example, the collaboration was critical in advising a nurse home visitor cited in the first paragraph of this paper on the policies governing the treatment of youth in foster homes and group homes, and a youth’s right to participate in their court case.

When legal representation attaches through the collaboration, both HELP: MLP and ICAC stay actively engaged with the client’s nurse home-visitor, with whom the client typically has the most long-standing and trusting relationship. With proper consent for information-sharing in place, nurse home-visitors support the lawyers’ roles by alerting the team to changes in the client’s goals, whereabouts, or services. The legal partners provide critical education and support on laws and practice so that the nurse home-visitors feel empowered to advocate on behalf of clients when there is an investigation or ongoing case. For example, in a case where the client was in foster care and wanted an independent living placement, ICAC provided guidance to the home-visitor before she testified that, in her professional judgment, the client could live independently with her baby. In a situation where the agency is investigating a client’s parenting, nurse home-visitors trained by lawyers can share their observations of the family and reinforce the duration and intensity of NFP’s support for the family, which tends to reassure risk-averse caseworkers.

In recent years, a new “family support fund” provides funds to help a family bridge a period of reduced income, which is almost unavoidable after the delivery of a baby due to the lack of guaranteed, paid parental leave in the state. The model requires that all housing and utilities requests are reviewed by HELP: MLP to ensure that there aren’t legal solutions or public benefits that can avert the crisis without a fund disbursement. Funded by individual donors and foundations, this fund allows our integrated partnership to bring its preventative role to bear much more effectively, as unpaid rent and utilities can be strategically addressed before legal ramifications and the corresponding stress they would cause. In 2022, the fund assisted 97 families with a range of requests including rental assistance, air conditioners, utility payments, groceries, furniture, and essential appliances.^18^ While the local child welfare agency has a “prevention” fund that can help families stabilize prior to the filing of a dependency petition, this fund cannot be utilized until there is an active child welfare report and investigation, which can cause family trauma through the threat of family separation. Because our fund is available to all families served by home-visiting programs, we can prevent the issues that might lead to a report before it happens and strengthen the positive relationship with our collaborative and the family.

Nurse home-visiting programs work with a broad swath of families, often from before their first child’s birth, and continue to serve the family through any child welfare involvement that may occur. With this framework, their model is simultaneously more effective at preventing government intervention, due to its early involvement, and at supporting reunification, because it does not withdraw its support when it is most needed, when government involvement is triggered. The integration of legal services and family support funds with these sustained family home-visiting programs only amplifies our collective ability to help families pursue their own goals, free of systemic intervention.

## II. Challenges and Perceived Barriers

While the potential impact of MLPs appears significant, practitioners and scholars have raised several challenges to the implementation of the MLP model in this context. Most frequently, practitioners raise the concern of mandated reporting laws as being barriers to the inclusion of families with existing or potential reports of suspected maltreatment. Of course, NFP nurses remain obligated to follow individual state mandated reporting laws, but they continue to have long-term and meaningful relationships with clients that are focused on advocacy, prevention, and education on everything from fire safety to appropriate child discipline at different stages of child development. Even with its mandated reporting obligations, the program still focuses on the relationship as the core of the intervention and does not find these goals to be fundamentally incompatible.

Many medical practitioners are trained to use child-welfare reporting as their first — or only — tool for addressing concerns about a family struggling to meet its basic needs, whereas nurse home-visitors partnered with lawyers receive training and resources on many more productive ways to support a family than calling in a child welfare report.^19^ While they respect their professional obligations, nurse home-visitors assess neglect much more narrowly on account of the many other tools they possess to help families meet their children’s needs.^20^

The legal partnership provides a foundation for engaging with parents and families that is broader than other stakeholders. In our partnership, based in Pennsylvania, where lawyers are not by default mandated reporters under state law, we are also able to leverage this difference in our professional responsibilities to support clients more fully.^21^ Nurse home-visitors are transparent with clients about the fact that they are mandated reporters, while their legal partners are not. If a client states an interest in talking to a lawyer without sharing the details of the concern with the nurse home-visitor, that referral is facilitated without judgment or professional friction. Clients are better served by being able to get candid legal advice without fear of reporting repercussions, especially in scenarios where there is intimate partner violence occurring in the family. Parents experiencing violence from their partners are often concerned — with good reason — that reporting violence occurring in their children’s presence will result in a child welfare report. While this happens infrequently, the ability to get legal advice via their nurse home-visiting relationship without disclosing the nature of the question to a mandated reporter, is a powerful tool offered by this partnership. Understanding the benefits of this approach is vital when starting an MLP involved with families and thinking through both how to strategically position the legal partner within the MLP and how to define the permissible cases to include parents potentially or currently at risk of a report of abuse or neglect.

The issue of defining the client when working with families is additionally raised as a barrier to the partnership, underscoring the adoption of a traditional child welfare mindset, which separates the child from the parent in terms of rights and legal representation. In contrast, the core values of home-visiting programs embody a radically empathetic strengths-based outlook and a belief that helping the parent helps the child — a philosophy that the MLP model can and should adopt. Our close partnership ensures that these values are embedded in our legal representations as well. When lawyers start from this foundational premise that the best interests of parents and children can be aligned and jointly advanced through legal representation, the threat of conflicts no longer looms as an insurmountable obstacle. As the adult in the household carries the legal responsibility — as a tenant, a claimant of public benefits, and an income-earner — they are formally the client in any representation, but there is a fundamental philosophical premise that the family is the unit we exist to support. We reject any contention that ethical lawyering is at odds with this philosophy. Rather than presuming an inherent conflict between the parent and child’s interests, as the family regulation system does, we pursue broad legal support for the family’s housing, income maintenance, and other needs that will contribute to their long-term stability beyond the family regulation system.

Finally, issues surrounding client information and aggregate data have been raised as an impediment to medical-legal partnerships related to child welfare, because an attorney for a parent or a child may not be able to disclose the outcome of a child welfare investigation or court appearance to their medical partner. Between our nurse home-visitors and lawyers, however, there is also a mutual professional understanding of the confidentiality obligations that adhere in each of our relationships. HIPAA and attorney-client privilege are well-understood limitations on our ability to share information freely. While we use a series of consent forms to ensure that home-visitors are permitted to share client information in making referrals, the lawyers operate more conservatively by asking clients for permission to share on any specific topic before disclosing information back to the nurse home-visitor. These protections are accepted as an inherent limitation of our partnership, and rarely cause challenges in meaningful inter-disciplinary engagement. Indeed, the focus on having the information sharing continually lodged in the client’s consent supports the model of client-centered lawyering. The trusting relationship between clients and nurse home-visitors is such that clients almost universally agree to the lawyer sharing information back with her nurse.

## III. Opportunities for Reform

Family regulation systems wield incredible power with their authority to intervene in a family’s life and remove children from their parents’ care. For decades, child and family advocates have sought reform of this system, highlighting the overrepresentation of families of color and the harm of removing children from their parents.^22^ From their inception, MLPs have promised to disrupt traditionally siloed practices by forging partnerships between medical practitioners and lawyers, historically antagonistic disciplines.^23^ MLPs focused on child welfare and family regulation system issues therefore present an opportunity for reframing the role of medical and legal professionals in families’ lives and for incorporating community-based and client-centered models into their practices. Increasing access to justice through MLPs allows for preventive advocacy that improves outcomes for children and families who would otherwise be impacted by the family regulation system.

Failing to focus on children and families with interactions with the family regulation system is a missed opportunity for reform, and potentially results in systematically dismissing a large percentage of families. The perceived barriers to focusing MLPs on these issues are reflective of the traditionally siloed practice in child welfare cases with a focus on removal versus reunification, as opposed to preventive services which prioritize family autonomy in the first instance. Moreover, there is already promising data that MLPs partnered with nurse home visiting programs could reduce involvement in the family regulation systems. In the U.S., the Nurse Family Partnership (NFP) reported promising outcomes from their home visiting program including a reduction in child abuse and neglect, which could be expanded through the resources available in the MLP addressing housing, benefits, and other civil legal issues.^24^ In Australia, researchers concluded that an NFP program reduced child protection system involvement for families who resided in an Aboriginal community in Central Australia and were serviced by the home visitors. ^25^

MLP leaders are uniquely positioned to propose reform in the family regulation system based on their expertise in the social and structural determinants of health, the individual and community level impact of preventive legal services, and evidence-based methods of improving child well-being outcomes to strengthen families. The legal partners have expertise in the role of civil legal services and the legal remedies available to families struggling with housing, benefits, or utilities issues.^26^ Their medical champions, exposed to successful interventions on behalf of struggling families, can be voices for change to address their own profession’s reliance on the family regulation system as a singular outlet for providers’ concerns for child health and well-being. Moreover, the MLP model of engaging research and policy reform are critical components of undoing the family regulation system. Collectively, these partnerships can forge a path for the two professions to undo some of the historic damage done, through their mutual goal of empowering and supporting children and families.

## Note

The authors have no conflicts to disclose.
